# Non-pharmaceutical Interventions for Hypertrophic Cardiomyopathy: A Mini Review

**DOI:** 10.3389/fcvm.2021.695247

**Published:** 2021-10-15

**Authors:** Miaomiao He, Jie Qiu, Yang Bai, Yan Wang, Mei Hu, Guangzhi Chen

**Affiliations:** ^1^Division of Cardiology, Department of Internal Medicine, Tongji Hospital, Tongji Medical College, Huazhong University of Science and Technology, Wuhan, China; ^2^Health Management Center, Tongji Hospital, Tongji Medical College, Huazhong University of Science and Technology, Wuhan, China

**Keywords:** hypertrophic cardiomyopathy, radiofrequency catheter ablation (RFCA), alcohol septal ablation, sudden cardiac death, next-generation sequencing

## Abstract

Hypertrophic cardiomyopathy is an inherited cardiovascular disease, and 70% of patients have left ventricular outflow tract obstruction. Ventricular septal myectomy has been the gold standard treatment for most patients with refractory symptoms. Due to higher mortality associated with medical facilities with less experience, alcohol septal ablation has been accepted as an alternative to conventional surgical myectomy. It offers lower all-cause in-hospital complications and mortality, which could be potentially more preferable for patients with serious comorbidities. In recent years, radiofrequency ablation, providing another option with reproducibility and a low risk of permanent atrioventricular block, has become an effective invasive treatment to relieve left ventricular outflow tract obstruction. Moreover, substantial progress has been made in gene therapy for hypertrophic cardiomyopathy. The principal objective of this review is to present recent advances in non-pharmaceutical interventions in hypertrophic cardiomyopathy.

## Overview of Hypertrophic Cardiomyopathy

Hypertrophic cardiomyopathy (HCM) is a hereditary cardiomyopathy. Epidemiological studies have shown that the prevalence in the general practice population is 1 in 500, but the rates are higher (1 in 200) if the genetic diagnosis is considered (1–3). Seventy percent of patients have hypertrophic areas involving the anterior ventricular septum and the basal segment of the anterior wall. Moreover, 70% of patients have mechanical impedance of the left ventricular outflow tract obstruction (LVOTO) (gradient ≥30 mmHg) in the resting or moving state, causing systolic anterior motion (SAM) of the mitral valve (4, 5). Recently, it has been reported that discrete sub-aortic membranes can cause LVOTO with or without SAM, which can be treated only with surgical intervention (6).

**Figure 1:**
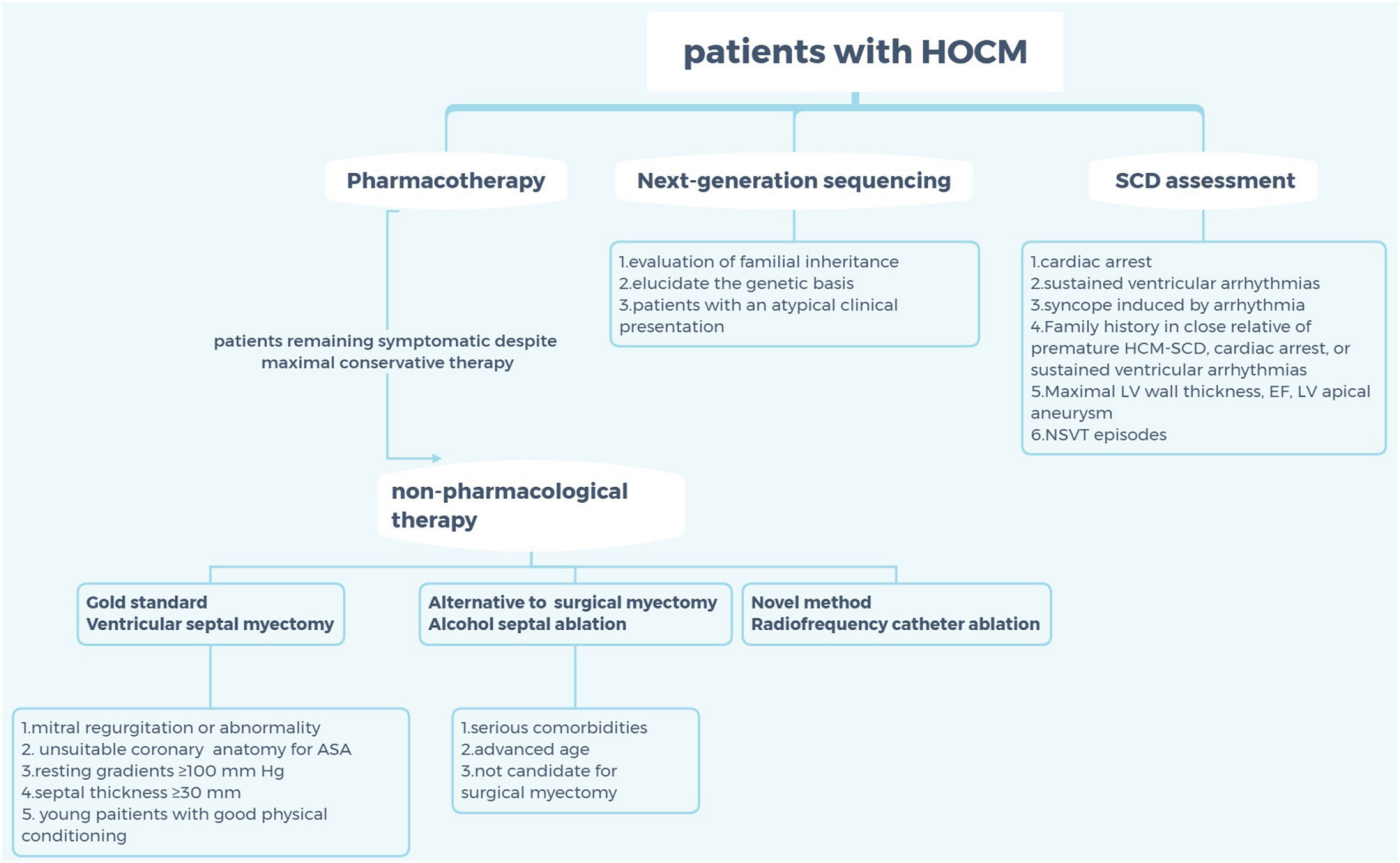
Criteria for selection of non-pharmaceutical interventions for patients with hypertrophic cardiomyopathy. HOCM, hypertrophic obstructive cardiomyopathy; SCD, sudden cardiac death; HCM, hypertrophic cardiomyopathy; EF, ejection fraction; LV, left ventricle; NSVT; non-sustained ventricular tachycardia; ASA, alcohol septal ablation.

The clinical manifestations of HCM are non-specific and diverse, which may result in a series of symptoms, including exertional dyspnea, fatigue, palpitations, dizziness, syncope, atypical chest pain, and sudden cardiac death (SCD) caused by ventricular obstruction (3, 7–9). Despite using the maximal pharmacologic dose and having no mitral valve and/or papillary muscle abnormalities, some patients with HCM still have obvious clinical symptoms; thus, approaches such as alcohol septal ablation (ASA) and ventricular septal myectomy (VSM) were recommended for these patients. Moreover, the role of radiofrequency catheter ablation (RFCA) in septal reduction therapy for HCM is increasingly being valued by interventional physicians in recent years. However, the choice of invasive treatment for patients with seemingly intermediate symptoms remains a debatable topic (Figure 1).

## Lifestyle Modifications

Persuading individuals with confirmed HCM to abandon competitive sports is a simple but effective way to decrease the incidence of SCD (10). However, exercise may directly improve diastolic dysfunction, as evidenced by electrocardiogram (ECG) findings and endothelial function, which are essential for regulating microcirculation (11, 12). In addition, there is scientific evidence that moderate-intensity exercise increases the exercise capacity in patients with HCM (13). Notably, the 2020 American College of Cardiology/American Heart Association (ACC/AHA) guidelines have encouraged patients with HCM to perform mild- to moderate-intensity recreational exercises to improve cardiorespiratory fitness.

## Release of the Obstruction

### VSM

For decades, septal myotomy, which involves the removal of a relatively small amount of muscle from the ventricular septum, has been the gold-standard treatment for most patients with HCM (14–16). Septal myectomy associated with mitral valve repair or replacement is an effective method for patients with HCM with severe mitral regurgitation (MR) or abnormality (17–21). However, the physiological function of the mitral valve cannot be preserved with mitral valve replacement and a clinical trial involving 2,107 cases showed that simple elimination of left ventricular outflow tract (LVOT) could reverse MR in 98% of patients (22, 23). What is even more remarkable is that almost all patients undergoing surgical resection will have left bundle branch block, and the mortality rate in small medical institutions is higher than that in large and experienced institutions. In a systematic study in 2003–2011, medical facilities with less experience were associated with higher mortality, longer hospital stays, and higher expenses (24). The advantages of myectomy are not matched by other minimally invasive procedures and drug therapy in cases of intrinsic abnormalities of the mitral valve, such as ruptured chordae tendinae or coexisting calcific or rheumatic mitral valve disease (25).

### ASA

Currently, ASA techniques have developed considerably, and ASA is accepted as an alternative to conventional surgical myectomy (26, 27). There is a large body of evidence that both procedures improve functional capacity, and there is no difference between ASA and VSM with regards to long-term all-cause mortality, cardiovascular mortality, or SCD (28, 29). The results of a large observational analysis of outcomes after ASA vs. VSM in patients with HCM suggested that ASA is associated with significantly lower all-cause in-hospital mortality and in-hospital complications and a higher rate of ventricular tachycardia, suggesting that ASA could be a better treatment procedure with fewer complications (30).

The selection criteria for ASA are generally based on anatomical features and increased operative risk advising against surgery. Not all patients with ventricular septal vascular anatomy are suitable for ASA. In 5%-15% of patients with HCM, ASA cannot be performed due to unsuitable coronary circulation anatomy and the presence of multiple submillimeter septal branches that are not accessible to the necessary armamentarium (31, 32). Invasive coronary angiography perioperatively assesses the information about the course and size of the coronary arteries and septal branches to determine whether the endovascular approach was adequate for ASA. Pre-procedural ECG-gated multidetector computed tomography imaging provides an advantage in localizing the appropriate septal branch by significantly diminishing the number of approached target vessels and non-ablated target vessels (33). It then provides a three-dimensional volume-rendering image of the coronary arteries and a two-dimensional image of the myocardium in diastole for the assessment of the spatial relationship between the septal branches and the target myocardial territories. Thus, the target septal artery could be selected, and the feasibility of ASA could be determined.

Pre-surgical implantation of temporary pacemaker has been suggested in patients undergoing ASA because up to 25% of these patients will have acute or subacute heart block and even require the implantation of a permanent pacemaker (34, 35). As expected, the rate of atrioventricular block requiring permanent pacemaker implantation was higher in younger patients (age ≤ 55 years) (36). Except for age, pre-ASA left bundle branch block, transient procedural high-grade block, post-ASA PR prolongation (≥68 ms), and new bifascicular block also were also predictors of complete heart block. These factors are reported to be important components of an 11-point clinical prediction tool, which thus allow the identification of high-risk patients who may benefit from additional monitoring and therapy (37). At present, the risk of ventricular tachyarrhythmia after ASA remains controversial. Alcohol inadvertently leaking from the target vessel could cause severe and extensive myocardial necrosis (38). Scars and islands of viable tissue after ASA are intertwined, which may cause arrhythmia (39). In a recent multicenter European study, appropriate implantable cardioverter defibrillator (ICD) shocks for ventricular tachycardia or fibrillation were eight-fold more common after ASA than after VSM (40). Notably, current monitoring following ASA using an implantable cardiac monitor could uncover clinically actionable events. A study followed up 56 patients with implantable cardiac monitor for 18 months, and the results showed that the overall cumulative rate of arrhythmic events was higher than that previously reported and that the most common arrhythmias following ASA were complete heart block and atrial fibrillation. However, whether ASA has a pathophysiological association with future development of atrial fibrillation is unknown. In this patient cohort, the cumulative rate of complete heart block detected by the implantable cardiac monitor within 18 months was 19%, which was mostly due to the injection of alcohol into septal perforators, resulting in injury to the His–Purkinje conduction system (41).

In August 2020, the ACC/AHA has released guidelines for the treatment of HCM, indicating that ASA may be less effective with high resting gradients (≥100 mmHg) and severe septal thickness (≥30 mm), and a study showed that the long-term all-cause and cardiac mortality rates in a 5-year follow-up are worse in patients with severe septal thickness (≥30 mm) (3, 42). As a consequence of these indications, ASA is potentially more preferable for patients with significant comorbidities that increase the risk of surgical myectomy, as the discomfort associated with surgery is avoided and the recovery time is reduced. Furthermore, the mid- and long-term arrhythmogenic potential of septal ablation needs to be appreciated, especially in older patients or those who, for different reasons, are not candidates for surgical myectomy.

### RFCA

RFCA, which is used to treat arrhythmia, has evolved over the last 20 years and features the formation of a high impedance on the surface of the myocardium and blockade of abnormal conduction of myocardial cells by high temperatures. Theoretically, radiofrequency (RF) energy can also be used to create myocardial injury in the ventricular septum to alleviate LVOTO. In recent years, the progress of methods, such as high-power and short-time ablation, active electrode cooling by saline irrigation, amplitude control ablation technology, and bipolar ablation technology, has made it feasible to create continuous lesions (43–49). Thus, treatment based on RF could be efficacious and has a longer-lasting effect.

Can RF be applied in the treatment of HCM? In 2004, Lawrenz and Kuhn (50) first reported a case of HCM treatment using RF. In this report, the patient was given procedural stimulation and marking of the His bundle using the Prucka mapping system. Then, 40 RF pulses were delivered to the right ventricular septum in a septal area of approximately 1 cm^2^. After resting and excitation, the LVOT gradient was significantly reduced. Even more surprising, the SAM symptom disappeared. This team further recruited 19 patients with HCM, and during these procedures, 14–40 RF pulses were delivered after marking the His bundle with a CARTO® catheter (Biosense Webster, Diamond Bar, CA, USA). According to the results, the LVOT gradient was reduced by 62% after resting and by 60% after provocation. Gadolinium-enhanced magnetic resonance imaging showed a late-enhanced area with a depth of 28 mm in the ablation area, but the thickness of the interventricular septum did not significantly decrease (51).

Some progress was made by Emmel and Sreeram (52), who performed RFCA in three children with HCM in 2005, two of whom had mitral valve malformations. Using the LocaLisa mapping system (Medtronic, Minneapolis, MN, USA) to mark the position of the His bundle, the operator performed ventricular septal ablation from the left ventricle through a retrograde and trans-aortic approach to generate three linear lesions between the left ventricular apical septum and the aortic valve. The LVOT decreased to <20 mmHg after 6 weeks and no complications occurred during the clinical follow-up.

In 2015, Shelke et al. (53) recruited five patients with HCM for RFCA. All patients were treated with the retrograde and trans-aortic approach using intracardiac echocardiography, the CARTO® system and EnSite NavX (Abbott, St. Paul, MN, USA) to mark the His bundle, left bundle branch, and Purkinje fibers during the operation. The ablation sites were the protruding parts of the ventricular septum in the transition area. It is worth noting that the ablation power and ablation time were reduced when the catheter moved close to the conduction system. After 1, 3, 6, and 12 months of follow-up, the LVOT of most patients continued to decrease, and no patient had atrioventricular block. Crossen et al. (54) reported the results of RFCA of the left ventricular septum, selecting transseptal access to the left atrium. However, the retrograde transaortic approach was applied when patients had an extraordinarily narrow outflow tract. The thickest part of the ventricular septum, which would be damaged by 120 s of continuous 50W RF energy, was marked on the NavX system. The LVOT in the stimulating and resting states in most patients decreased continuously by 85% after 12 months and the New York Heart Association class increased by at least one level. Cooper et al. (38) reported the first RFCA guided by CARTOSound® (a combination of echocardiography and CARTO®; Biosense Webster), in which lesions were placed over the SAM-septal contact area and the energy power was 50–60 W with a maximum temperature of 60°C. The patient's resting LVOT gradient decreased 51.9 mmHg, the Valsalva or exercise-induced gradient decreased to 70.2 mmHg, and the New York Heart Association class improved from III to II/I.

RFCA can produce predictable, discrete lesions, with a clear boundary between the scar and healthy surrounding muscles, which, in principle, can reduce the tendency of arrhythmia. The three-dimensional mapping system has been applied in RFCA to mark the heart conduction system and avoid damage as much as possible. A previous study showed that the incidence of conduction system damage could be reduced by decreasing the RF energy and time ablation. For a person who cannot choose ASA and has a contraindication for myectomy, RFCA provides another option with reproducibility and a low risk of permanent atrioventricular block. Although there was no recommendation in the guidelines, RFCA may be best for some patients with poor physical conditions and mildly thickened interventricular septum. In addition, repeated RFCA of the ventricular septum is feasible (54).

However, the use of RFCA in treatment of HCM is still under investigation and development, and many studies have provided evidence that RFCA increases the risk of abnormally increased LVOTO in HCM patients in the late-stage due to tissue edema, which needs to be considered preoperatively. In addition, adequate anticoagulation should be used during the operation, and minimum liquid flushing of the tip of the catheter and diuretics could avoid liquid overload, which can improve the safety of the operation. Prolonged operation of the left ventricular catheter may cause transient atrial fibrillation, and abnormal mitral valve papillary muscle attachment may play an important role in the occurrence of LVOTO, which should be carefully evaluated before surgery (for a summary of operation, see Table 1).

**Table 1:** Advantages and disadvantages of different operative management.

	**Ventricular septal myectomy**	**Alcohol septal ablation**	**Radiofrequency catheter ablation**
Advantages	• Gold standard • Treatment of mitral valve repair or replacement	• Lesser iatrogenic trauma • Alternative to surgical myectomy • Shorter stay in the hospital • Lower cost	• Predictable lesions • Avoiding damage to conduction system • Reproducibility
Disadvantages	• Relatively higher mortality rate • Longer stay in the hospital • Higher expenses	• Limitation of vascular anatomy • Occurrence of heart block • Possibility of myocardial necrosis	• Risk of abnormally increased left ventricular outflow tract obstruction • Hemorrhage due to anticoagulation • Occurrence of transient atrial fibrillation </List>

### Transcatheter Mitral Valve Repair

In patients with HCM, LVOTO develops during left ventricular ejection in mid-systole due to SAM. The elongation and enlargement of anterior mitral leaflet and posterior mitral leaflet lengths, common in HCM patients, is an important morphological abnormality responsible for SAM syndrome (55). Transcatheter mitral valve repair, plicating the valve leaflets and preventing SAM of the valve, could offer a new treatment modality for the HCM patients. In 2017, Sorajja et al. (56) firstly treat the HCM patients using percutaneous mitral valve plication as a primary therapy. These patients are not optimal candidates for VSM due to their advanced age and frailty. In the postprocedural follow-up, the SAM of the valve and LVOT gradient of most patients was reduced, and symptom was improved by at least 1 New York Heart Association functional class without major adverse clinical events. Interestingly, Coylewright et al. (57) reported a case about HCM treatment using percutaneous edge-to-edge repair device. Transcatheter mitral valve repair with MitraClip (Abbott Laboratories, Abbott Park, IL, USA) was performed with one clip via a transseptal approach. At the postoperative evaluation, MR and SAM of the anterior mitral leaflet was reduced. Transcatheter mitral valve repair may be an effective therapy for patients with degenerative MR and concomitant LVOT obstruction. Further basic science research and eventual randomized clinical trials are needed to make claims of efficacy and safety for this novel treatment.

## SCD Assessment and Prevention

SCD affects at least 0.2% of the global population and is the most common cause of fatal ventricular arrhythmia in young people and athletes (58). The 2020 ACC/AHA guidelines combine several clinical markers into a risk stratification algorithm to identify populations at high risk who should promptly undergo ICD and other prevention programs that can effectively terminate ventricular tachycardia and fibrillation such as those with history of cardiac arrest, syncope suspected by arrhythmia, etc., (59–62). The SHIFT model may provide prognostic value for ICD implantation for the primary prevention of SCD (63). All patients with diagnosed or suspected HCM should be evaluated every 1–2 years, especially those who may be eligible for ICD indications (58). In 2013, O'Mahony C et al. (66) proposed the HCM Risk-SCD Calculator, a new HCM SCD clinical risk prediction model derived from a retrospective multicenter longitudinal cohort study, which lies in the shift from relative risk assessment to absolute risk assessment (64, 65). However, this model also has shortcomings; for example, it cannot be applied to pediatric patients or elite or competitive athletes younger than 16 years. Moreover, late gadolinium enhancement has emerged as an important predictor for SCD, and the incidence rate of SCD/aborted SCD in patients with HCM with positive late gadolinium enhancement is greater than that in patients with negative late gadolinium enhancement (67).

Patients with a risk of 4% in the HCM risk calculator and at least two major risk factors (left ventricular apical aneurysm or end-stage HCM) receive ICD treatment (66, 68). Moreover, a recent paradigm has suggested that ICD therapy responsible for a substantial decrease in overall HCM-related mortality is independent of patient age (69). It is imperative to make ICD decisions for younger HCM patients at high risk for SCD; however, it has low pacing probability (66). Additionally, there is no evidence that drug therapy has a protective effect against SCD caused by malignant ventricular arrhythmia in individuals with HCM (70). Therefore, the use of drugs to prevent SCD in asymptomatic patients with HCM is not recommended.

## Gene Therapy

**Figure 2:**
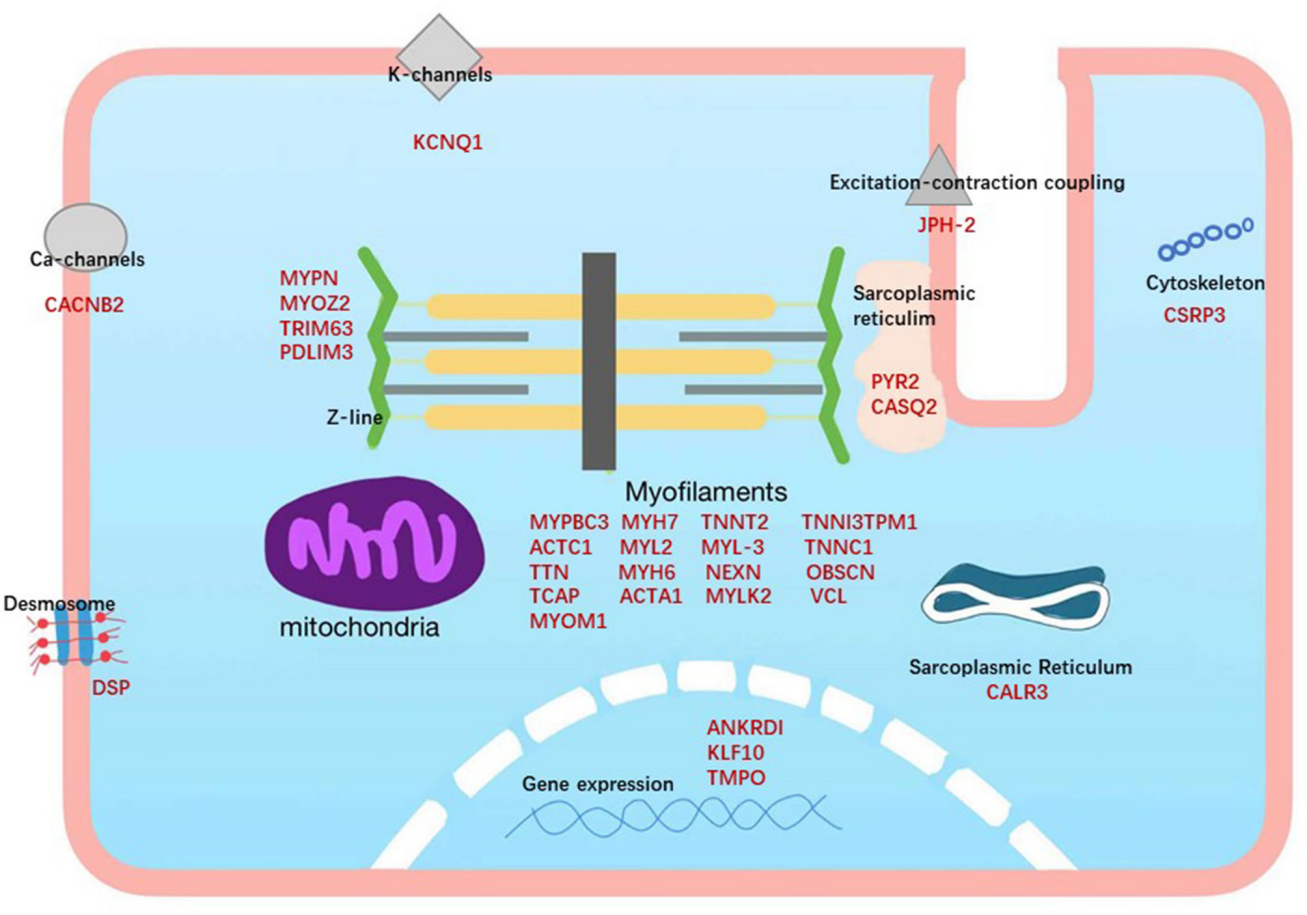
Subcellular locations of the proteins encoded by the genes associated with hypertrophic cardiomyopathy. HCM genes are shown in red.

HCM is an inherited disease and almost always results from mutations in multiple protein-coding genes, modulation by non-coding RNAs, and perturbations in gene networks. Approximately 35%−60% of patients carry pathogenic mutations in the autosomal dominant sarcomere protein gene, which is related to 1,400 mutations in more than 11 different genes (71–75). Among these HCM genes, only 8 genes have definitive evidence of disease association (MYBPC3, MYH7, TNNT2, TNNI3, TPM1, ACTC1, MYL2, and MYL3), 3 genes have moderate evidence (CSRP3, TNNC1, and JPH2), and 22 have limited or no evidence (TTN, KLF10, MYPN, etc.) (Figure 2) (76). Current research has found that Filamin C (FLNC) plays a prominent role in HCM. The dysfunction of FLNC could interfere with the dimerization and folding of the protein, leading to aggregate formation, which is detrimental to muscle function, as found in HCM. In addition, pathogenic mutations in the sarcomere genes MYH7 and MYBPC3 explain 60–70% of observed clinical cases, and they could exist in biallelic inheritance or more complex variants, including CNVs and *de novo* mutations (77).

Compared with familial HCM, individuals with non-familial HCM have late-onset and less severe disease (78–80). The correlation between genotype and phenotype is not consistent because of the complex genetic basis, epigenetics and other mechanisms for regulating gene expression, environmental conditions such as non-sarcomere genetic variations, and more importantly, the overlap among several disease gene groups (81–85). These factors make genetic testing and result interpretation particularly challenging.

Next-generation sequencing (NGS) is an effective method for measuring and analyzing gene mutations in HCM (86). The 2020 ACC/AHA guidelines recommended next-generation sequencing for diagnosing HCM, suggesting that cascade screening should be performed in family members at risk in order to prevent or treat appropriately (3, 87). Periodic examination with echocardiogram and ECG is encouraged for all first-degree family members (88); meanwhile, individuals with negative genotypes do not need routine ECG or echocardiogram (68, 81). In a retrospective analysis of adult and pediatric splotch mutation carriers identified during family screening, results showed that approximately 50% of splotch mutation carriers develop HCM over 15 years and that independent predictors of HCM development were male sex and abnormal ECG (89). This study indicates that the timing of follow-up could be tailored to the splotch mutation carriers, male sex, and abnormal ECG, rather than age. Whole-genome sequencing could identify gene variants in 9% of HCM with negative next-generation sequencing results, which also had shortcomings in discovering a large number of variants of uncertain clinical significance (90, 91). Moreover, multiple gene variants have been identified in 0.4% of the patients and are correlated with adverse cardiac events (92, 93). Children with a positive phenotype are at greater risk of serious clinical manifestations and cardiac events and clinical screening at an earlier age is currently recommended (94, 95).

Gene therapy could be considered, particularly genome editing, exon skipping, allele-specific silencing, and RNA trans-splicing, most of which are in the stage of molecular research or animal experimentations. Successful gene replacement with the delivery of functional MYBPC3 cDNA mediated by adeno-associated vector 9 in human cardiomyocytes derived from embryonic stem cells proved that gene replacement is a feasible therapeutic option (96). Adenovirus vectors with high safety and feasibility have been proven in published studies and are frequently used as carriers; meanwhile, genetic correction using CRISPR technology eliminates the electrophysiological abnormalities in the patient's induced pluripotent stem cells (97, 98). With these advances, gene therapy could be a viable treatment option for HCM, especially for its severe forms.

In summary, research on HCM has made great progress in recent years; nevertheless, some questions and challenges remain to be solved by further research. After decades of continuous efforts, ASA has been accepted as an alternative to conventional surgical myectomy and is potentially more attractive for patients with significant comorbidities that increase the risk of surgical myectomy, as the discomfort associated with surgery is avoided and the recovery time is reduced. RFCA is a novel method for relieving LVOTO, and preliminary result are promising; however, further clinical trials are still necessary. Gene therapy is also particularly attractive option for HCM, and the prospect of clinical therapy is on the horizon. New therapies that target the causes of HCM or slow the progression of the disease are urgently needed.

## Author Contributions

GZC and MH conceived of the presented idea. JQ and YW developed the theory. MMH and GZC wrote the initial draft of the manuscript. All authors contributed to the article and approved the submitted version.

## Funding

This work was supported by grant from the National Natural Science Foundation of China (No. 82070383).

## Conflict of Interest

The authors declare that the research was conducted in the absence of any commercial or financial relationships that could be construed as a potential conflict of interest.

## Publisher's Note

All claims expressed in this article are solely those of the authors and do not necessarily represent those of their affiliated organizations, or those of the publisher, the editors and the reviewers. Any product that may be evaluated in this article, or claim that may be made by its manufacturer, is not guaranteed or endorsed by the publisher.
